# Periodontitis Salivary Microbiota Exacerbates Murine Rheumatoid Arthritis via Gut Dysbiosis and Immune Dysregulation

**DOI:** 10.1096/fj.202502610R

**Published:** 2025-12-01

**Authors:** Ruiyang Ge, Rong Liu, Yingying Zhou, Ziyao Zhuang, Haowei Mao, Wenzheng Liao, Lei Han, Wenrong Yang, Fuhua Yan, Di Cui

**Affiliations:** ^1^ Department of Periodontology, Nanjing Stomatological Hospital, Affiliated Hospital of Medical School, Institute of Stomatology Nanjing University Nanjing China; ^2^ Guiyang Hospital of Stomatology Guiyang China; ^3^ Department of Orthodontics, Nanjing Stomatological Hospital, Affiliated Hospital of Medical School, Institute of Stomatology Nanjing University Nanjing China; ^4^ Shenzhen Hospital, Southern Medical University Shenzhen China; ^5^ School of Life and Environmental Sciences, Centre for Sustainable Bioproducts Deakin University Waurn Ponds Geelong Victoria Australia

**Keywords:** immune dysregulation, microbiota transplantation, oral‐gut‐immune axis, periodontitis, rheumatoid arthritis

## Abstract

The mechanistic link between periodontitis (PD) and rheumatoid arthritis (RA) remains poorly understood. This study aimed to investigate whether PD‐associated salivary microbiota exacerbates RA via an “oral‐gut‐immune axis.” We performed 16S rRNA sequencing and untargeted metabolomics on saliva from healthy controls and patients with PD, RA, or both (PD‐RA). Salivary microbiota from PD patients or healthy controls were transplanted into collagen‐induced arthritis (CIA) mice. Clinically, PD‐RA patients exhibited enrichment of periodontal pathogens (e.g., *Porphyromonas* sp., *Prevotella* sp.) and pro‐inflammatory lipid metabolites. In vivo, transplantation of PD microbiota into CIA mice significantly worsened arthritis, evidenced by higher clinical scores (3.5 ± 0.5 vs. 2.3 ± 0.4, *p* < 0.05) and greater joint destruction. Mechanistically, PD microbiota colonized the gut, impaired intestinal barrier integrity, and induced systemic immune dysregulation, characterized by an increased splenic Th17/Treg cell ratio and elevated serum IL‐6 and C‐reactive protein. These changes were associated with altered gut microbial metabolism, including reduced short‐chain fatty acids and enhanced arachidonic acid pathways. Our findings delineate a pathogenic oral‐gut‐immune axis in which PD microbiota aggravates RA, underscoring the importance of integrated oral and systemic disease management.

## Graphical abstract

Salivary multiomics from healthy, PD, RA, and PD‐RA cohorts identified PD−/RA‐linked microbial and metabolic signatures. We then transplanted salivary microbiota from a PD donor or a healthy donor into collagen‐induced arthritis mice. PD microbiota colonized the gut, reshaped communities, weakened epithelial tight junctions, and provoked systemic immune imbalance with higher serum IL‐6/CRP. Consequently, arthritis scores and bone erosion increased. Findings delineate an oral–gut–immune axis whereby PD microbiota aggravate arthritis and highlight integrated oral–systemic management.

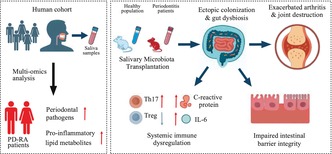

## 1 Introduction

Periodontitis (PD), frequently initiated by keystone pathogens such as *Porphyromonas gingivalis* ( *P. gingivalis* ), affects approximately 10% of adults globally [1]. Rheumatoid arthritis (RA) is a systemic autoimmune disorder characterized by chronic synovitis and progressive bone erosion, resulting in significant morbidity [2, 3]. Despite extensive research, its pathogenesis remains incompletely defined. Current therapies targeting cytokines or immune checkpoints are limited by infection risk and immune suppression [4]. Epidemiologic studies indicate that patients with RA have a substantially higher prevalence of moderate‐to‐severe PD, exceeding 70%, and those with PD exhibit elevated RA disease activity [5, 6, 7, 8, 9]. These observations suggest that local periodontal infections may drive systemic immune activation in RA, supporting a bidirectional relationship between the two diseases.

The “oral‐gut axis” describes a pathway wherein ingested oral microbiota survive gastrointestinal transit, colonize the gut, and alter the local microbial ecology, which in turn compromises intestinal barrier integrity and modulates systemic immune responses, thereby linking oral dysbiosis to extra‐oral diseases [10]. In recent years, the concept of the “oral‐gut axis” has offered new insights into the association between periodontitis and systemic diseases. Humans swallow approximately 1.5 L of saliva daily, containing numerous oral microbes. Under healthy conditions, the gastrointestinal barrier prevents the translocation of most ingested microbiota. However, in disease states, alterations in oral microbiota can disrupt gut microecology and elicit systemic effects. Murine studies have shown that salivary microbiota from patients with PD can induce gut dysbiosis and compromise intestinal barrier integrity [11, 12]. Similarly, gut barrier dysfunction is reported in RA [13, 14]. Even before clinical onset, RA is associated with increased gut permeability and microbial dysbiosis, which weakens mucosal defenses, activates mucosal immunity, and promotes the migration of pro‐inflammatory cells, such as Th17 cells and activated macrophages, to the periphery and joints. These findings suggest that the dysbiotic oral microbiota in PD may influence the gut via saliva and trigger immune responses in susceptible hosts, potentially representing a mechanistic link by which PD exacerbates RA pathogenesis.

Microbiota‐mediated autoimmune mechanisms have gained attention in the context of PD and RA. A classic hypothesis suggests that the periodontal pathogen *P. gingivalis* expresses a unique peptidylarginine deiminase (PAD) enzyme, which citrullinates bacterial and host proteins. This modification generates neoantigens that breach immune tolerance and elicit RA‐specific autoantibody responses [2]. Moreover, PD and RA share common inflammatory pathways [15, 16, 17]. Cytokines such as interleukin‐6 (IL‐6), tumor necrosis factor‐α (TNF‐α), and Th17‐mediated osteoclastogenesis play essential roles in the pathogenesis of both diseases. However, direct evidence linking PD‐associated oral microbiota to RA through gut microbial‐metabolic pathways remains limited.

Based on these observations, this study aimed to elucidate the microbial and metabolic mechanisms underlying the PD‐RA interplay through combined clinical and animal experiments. Multiomics analyses were first conducted on salivary microbiota and metabolites in healthy individuals, patients with PD, RA, and RA with PD, evaluating microbial taxa and metabolic pathway alterations, and correlating them with disease phenotypes. Next, salivary microbiota were transplanted from patients with PD into collagen‐induced arthritis (CIA) mice to assess arthritis progression and immune responses, thereby testing the pathogenic role of PD‐related oral microbiota in exacerbating RA. Colonization by PD‐associated bacteria is hypothesized to disrupt host gut microbiota and metabolism, disrupt intestinal barrier integrity, and induce pro‐inflammatory immune responses (such as Th17 expansion), aggravating RA. Our findings aim to provide mechanistic insight supporting the integrated management of both diseases.

## 2 Materials and Methods

### 2.1 Clinical Study Participants and Saliva Sample Collection

A total of 40 human participants were enrolled and divided into four groups (*n* = 10 per group) based on periodontal and RA status: (1) healthy controls (systemically and periodontally healthy), (2) PD group (systemically healthy with generalized severe PD), (3) RA group (patients with RA and periodontal health), and (4) PD‐RA group (patients with RA and severe PD). The demographic and clinical characteristics of the participants are summarized in Table 1. The groups were generally balanced in terms of sex and age distribution. PD was diagnosed according to the 2018 American Academy of Periodontology (AAP)/European Federation of Periodontology (EFP) classification, and participants with generalized Stage III, Grade B PD (indicating advanced attachment/bone loss and moderate progression) were included. RA was diagnosed based on the 1987 American College of Rheumatology (ACR) revised criteria, requiring at least four clinical features (such as morning stiffness ≥ 1 h, symmetric arthritis, and positive rheumatoid factor). Participants who had received periodontal treatment within the past 6 months or had systemic diseases (such as diabetes, gastrointestinal diseases, or immunodeficiencies), or recent antibiotic or anti‐inflammatory use were excluded. All participants provided informed consent, and the Ethics Committee of Nanjing University Medical School Affiliated Stomatology Hospital approved the study (Approval No. NJSH‐2022NL‐059).

**TABLE 1:** Sex and mean age distribution across groups.

Group	*N*	Male, *n*	Female, *n*	Age (years), mean ± SD	Systemic condition
Healthy control	10	4	6	36.6 ± 11.1	Healthy
Severe periodontitis	10	3	7	41.5 ± 8.8	Healthy
Rheumatoid arthritis	10	4	6	48.5 ± 11.3	No other systemic disease
severe periodontitis + Rheumatoid arthritis	10	3	7	44.8 ± 9.8	No other systemic disease

### 2.2 Saliva Sample Collection

Saliva was collected in the morning. Participants were instructed to avoid eating or drinking after brushing their teeth the night before, and to refrain from brushing or rinsing on the morning of collection. During collection, participants sat upright with heads slightly tilted forward, allowing unstimulated saliva to drip passively from the lower lip into sterile tubes. Swallowing and forceful suction were avoided to minimize gingival bleeding contamination. Saliva was intermittently collected (participants drooled into the tube every ~2 min) over ~10 min until ≥ 3 mL of whole saliva was obtained. The collected saliva was immediately placed on ice (4°C), centrifuged at 1000 rpm for 10 min, and the supernatant was transferred to a new tube. An equal volume of sterile 20% glycerol‐PBS cryoprotectant solution was added, mixed thoroughly, then rapidly frozen in liquid nitrogen and stored at −80°C until further analysis.

### 2.3 Establishment of the CIA Model

Twenty‐four male dilute brown Agouti (DBA)/1 Jackson Laboratory (1 J) mice (6–8 weeks old; purchased from Shanghai SLAC Laboratory Animal Co. Ltd.) were used to establish the CIA model. Following a 1‐week acclimatization, mice were randomly assigned (*n* = 6 per group) using a random number table to: normal control (Con_PBS), CIA + PBS group (CIA_PBS), CIA + PD microbiota group (CIA_P), and CIA + healthy microbiota (CIA_H). CIA was induced under specific‐pathogen‐free conditions using standard protocols. Bovine type II collagen (Chondrex, Woodinville, WA 98072, USA) was emulsified with an equal volume of complete Freund's adjuvant (Chondrex, Woodinville, WA 98072, USA). On day 0, 240 μL of this emulsion was injected at multiple sites (subcutaneously at the tail base and back, and into both hind footpads) for primary immunization. On day 21, a booster was administered: type II collagen emulsified with incomplete Freund's adjuvant (100 μL) (Chondrex, Woodinville, WA 98072, USA) was injected near the initial site. Control mice (Con_PBS) received PBS on the same schedule.

Postinduction, mice were monitored daily for joint redness and swelling. Arthritis severity was assessed every 3 days beginning on day 2 using a standard scoring system: 0 = no redness or swelling; 1 = mild swelling; 2 = moderate swelling extending to the tarsal joint; 3 = severe swelling; 4 = severe swelling with ankylosis. Each hind paw was scored (0–4), and scores from all limbs were summed to yield a cumulative arthritis score per mouse.

### 2.4 Microbiota Gavage

Gavage with human salivary microbiota began on day 22, 1 day after the booster. Saliva samples from a PD patient and a healthy donor (one each) were thawed in a 37°C water bath, centrifuged at 3300 g for 10 min at 4°C, and the pellets resuspended in sterile PBS. Mice in the CIA_P and CIA_H groups were gavaged orally with 200 μL of the respective bacterial suspensions, while the Con_PBS and CIA_PBS groups received sterile PBS. Gavage was performed every other day for 6 weeks.

### 2.5 Sample Collection and Analysis

At the end of the 6‐week gavage period (approximately day 63), all mice were sacrificed under isoflurane anesthesia, and the following samples were collected: (a) After final arthritis scoring, ~1 mL of blood was collected from the orbital sinus into sterile tubes. After clotting at room temperature for 1 h, samples were centrifuged at 3000 rpm for 10 min at 4°C. The serum was separated and stored at −80°C for inflammatory cytokine analysis. (b) Under sterile conditions, spleens were removed and placed in Roswell Park Memorial Institute Medium (RPMI)‐1640 medium. Single‐cell suspensions were prepared by mechanical dissociation for flow cytometric analysis of T‐cell subsets. (c) Both hind limbs were excised approximately 1 cm above the ankle joints for subsequent tissue analysis. The left hind ankle was fixed in 4% paraformaldehyde for bone morphology and histopathological evaluation. In contrast, the right hind ankle was snap‐frozen in liquid nitrogen and stored at −80°C for micro‐CT scanning to assess bone destruction. (d) Cecal contents were collected into sterile tubes, snap‐frozen in liquid nitrogen, and stored at −80°C for 16S rRNA sequencing of gut microbiota. (e) A ~1 cm segment of distal colon was excised; one portion was fixed in 4% paraformaldehyde for histology, and the remainder was placed in RNA stabilization solution and frozen for later evaluation of intestinal barrier integrity and inflammation‐related gene expression.

### 2.6 Enzyme‐Linked Immunosorbent Assays (ELISAs)

Serum levels of inflammatory markers IL‐6 (Neobioscience Technology Co. Ltd., Shenzhen, China) and CRP (Elabscience Bionovation Inc., Houston, Texas, USA) were quantified using commercial enzyme‐linked immunosorbent assay (ELISA) kits per the manufacturers' protocols.

### 2.7 Flow Cytometry Analyses

Splenic mononuclear cells underwent red blood cell lysis, followed by flow cytometric analysis of Treg and Th17 cell proportions among CD4^+^ T cells. Mouse Treg Cell Staining Kit and Mouse Th17 Cell Staining Kit (Liankebio, Hangzhou, China) were used. For Treg analysis, cells were surface‐stained with anti‐CD4‐fluorescein isothiocyanate (FITC) and anti‐CD25‐allophycocyanin (APC) antibodies, fixed and permeabilized, and then stained intracellularly with anti‐forkhead box P3 (Foxp3)‐phycoerythrin (PE) antibodies. For Th17 analysis, cells were stimulated with a phorbol 12‐myristate 13‐acetate (PMA)/ionomycin mixture (250×) and a brefeldin A/monensin (250×) for 6 h, surface‐stained for CD4, fixed, permeabilized, and stained intracellularly with anti‐IL‐17A‐PE. A minimum of 100 000 lymphocyte events were acquired per sample on a flow cytometer, and FlowJo software was used to quantify CD4^+^Foxp3^+^ Treg and CD4^+^IL‐17A^+^ Th17 cells within the CD4^+^ T cell population.

### 2.8 Micro‐Computed Tomography (Micro‐CT)

The hind limbs were imaged by SkyScan 1276 Micro‐CT (Bruker, Germany). Three‐dimensional reconstruction of the hind limbs was performed with Data Viewer software, and bone parameters were analyzed using CTAn software.

### 2.9 Histological Changes

Mice were intraperitoneally injected with pentobarbital sodium and then euthanized. The joints of rats or the paws of mice were fixed in 10% formalin, embedded in paraffin, and sliced into 4 μm thick sections. H&E staining was then performed to assess the histological grading of the disease.

### 2.10 16S rRNA Sequencing

Total DNA was extracted from both clinical saliva and mouse cecal samples. The V3‐V4 hypervariable region of the bacterial 16S rRNA gene was polymerase chain reaction (PCR)‐amplified and purified for high‐throughput sequencing using an Illumina platform. Raw reads were processed with Cutadapt software (Max Planck Institute for Developmental Biology, Tübingen, Germany) to remove primers, then quality‐filtered, denoised, and merged using the Divisive Amplicon Denoising Algorithm 2 (DADA2) within Quantitative Insights Into Microbial Ecology 2 (QIIME2) (version 2019.4). After chimera removal, high‐quality amplicon sequence variants (ASVs) and an abundance table were obtained. Taxonomic classification of ASVs was performed using the Greengenes database (Release 13.8) via the QIIME2 classifier. Singletons (reads appearing only once across all samples) were excluded to generate the final species abundance profiles.

Alpha diversity was assessed using QIIME2, including species richness and diversity indices. Rarefaction curves confirmed adequate sequencing depth. Group comparisons of alpha diversity were conducted using the Kruskal–Wallis rank‐sum test. Beta diversity was analyzed using Bray‐Curtis distance matrices, with PCoA and nonmetric multidimensional scaling (NMDS) used for visualizing intergroup differences. Statistical significance in community composition was evaluated by the Adonis permutational ANOVA test (PERMANOVA). Furthermore, LEfSe was applied to identify taxa with significant differential abundances between groups; taxa with Linear Discriminant Analysis (LDA) scores > 2 and *p* < 0.05 were considered discriminatory.

### 2.11 Untargeted Metabolomics Analysis

Human saliva and mouse cecal contents underwent untargeted metabolomic analysis. For each sample, 200 μL of sample was mixed with a prechilled methanol‐acetonitrile solution to precipitate proteins and extract metabolites. After centrifugation, the supernatant was vacuum‐dried and reconstituted in 70% methanol. Metabolites were analyzed using ultra‐high‐performance liquid chromatography coupled with quadrupole Orbitrap mass spectrometry (MS) (UHPLC‐Q Exactive MS, Thermo Fisher Scientific Inc., Waltham, Massachusetts, USA) in both positive and negative ion modes. Raw MS data were converted to an open format (mzXML) using ProteoWizard, and then processed with XCMS for peak detection and retention time correction. Metabolites were identified by comparison with an in‐house standard library and public databases (such as Human Metabolome Database [HMDB] and Metabolite and Chemical Entity Database [METLIN]) for both MS^1^ and MS^2^ spectra.

The resulting data matrix was Pareto scaled and analyzed using Soft Independent Modeling of Class Analogy (SIMCA) software. Principal component analysis (PCA) identified sample distributions and outliers. The OPLS‐DA was used to model metabolic differences between groups. The S‐plots from the OPLS‐DA model, combined with univariate *t*‐tests and multiple testing corrections, were used to identify significant metabolites. These were further analyzed through Kyoto Encyclopedia of Genes and Genomes (KEGG) pathway enrichment to explore the functional significance of metabolic alterations.

### 2.12 Statistics

Data are shown as the mean ± SD in bar graphs. Differences between 2 groups were evaluated with Student's *t* test or the Wilcoxon test. For comparing > 3 groups, a 1‐way analysis of variance or Kruskal–Wallis test was performed. Statistical analyses were performed with Prism 9 (GraphPad Software Inc).

## 3 Results

### 3.1 Differences in Salivary Microbiota Structure Between Patients With PD and RA

**FIGURE 1:**
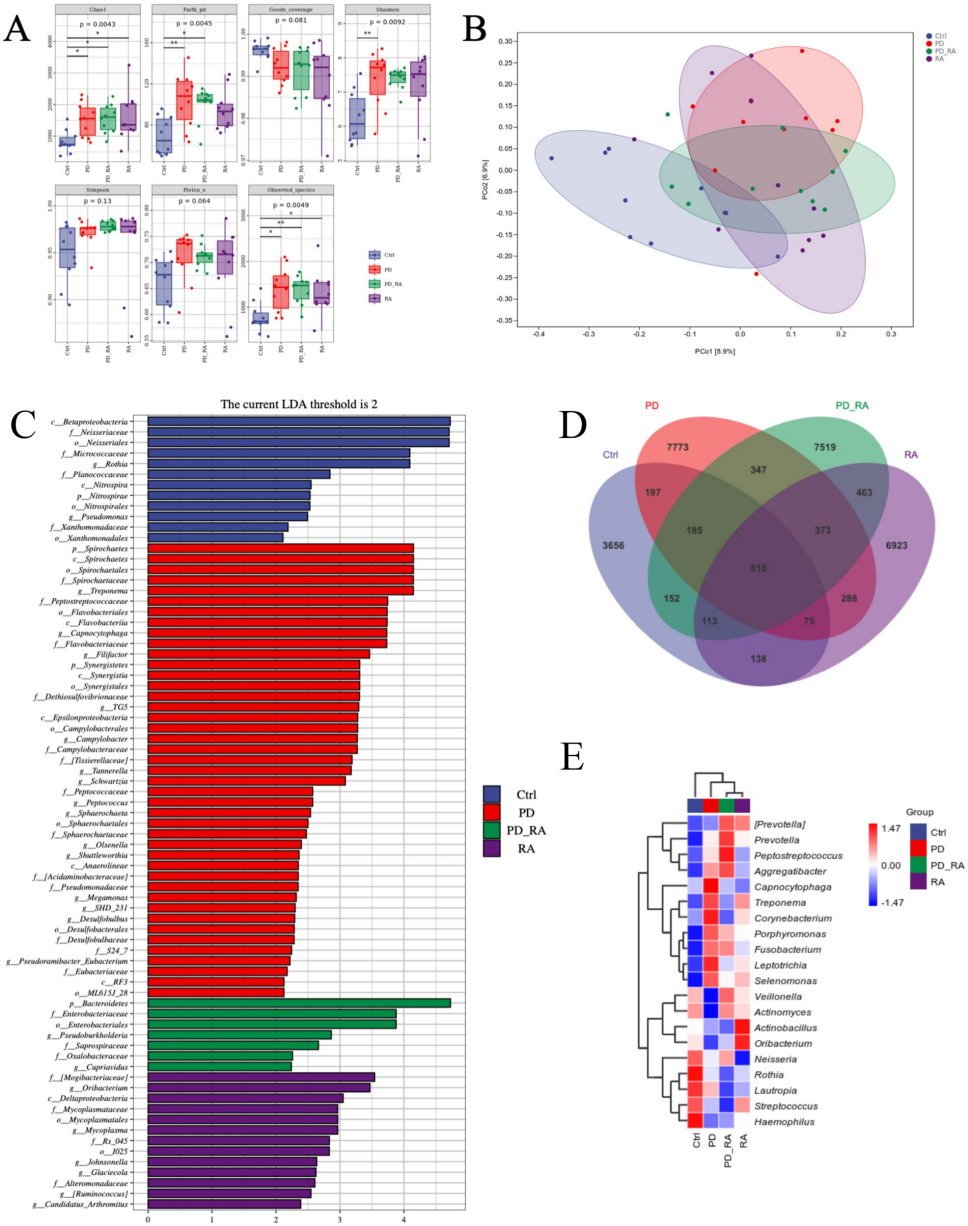
Salivary microbiota diversity and composition differences among healthy, periodontitis, RA, and RA + PD patients. (A) Alpha diversity of salivary microbiota: Each boxplot shows the median, interquartile range, and whiskers (minimum and maximum values). (B) PCoA scatter plot of salivary microbiota beta diversity, showing the distribution of samples from healthy control, periodontitis (PD), RA, and PD‐RA groups. (C) LDA score bar chart of significantly different genera between groups (identified by LEfSe). (D) Venn diagram showing the number of shared and unique amplicon sequence variants (ASVs) among the groups. (E) Heatmap of the relative abundance of the top 30 genera in salivary microbiota. (Ellipses in panels represent 95% confidence intervals. *: *p* < 0.05; **: *p* < 0.005).

The 16S rRNA gene sequencing of saliva samples from 40 participants yielded 2.58 million high‐quality reads (average: 64 554 per sample), ensuring sufficient depth for diversity analysis. α‐diversity assessment revealed significant intergroup differences across diversity indices (control, PD, RA, PD‐RA) (Figure 1A). Kruskal**–**Wallis comparisons of the Chao1 index, Faith's PD index, Observed_species index, Shannon index, Simpson index, Pielou_e evenness, and Good's coverage identified significant differences. Chao1 and Observed_species indices were significantly higher in the PD and PD‐RA groups in controls (*p* < 0.05), indicating increased species richness. Shannon diversity was also elevated in the RA and PD‐RA groups, with the PD‐RA versus control difference reaching significance (*p* < 0.05), suggesting increased microbial diversity in patients with RA, especially those with PD. Faith's PD index, reflecting phylogenetic diversity, was highest in the PD group and significantly greater than in controls (*p* < 0.05), indicating elevated phylogenetic richness in PD. Pielou's evenness and Simpson's index trended higher across disease groups; however, they did not reach significance, suggesting that while richness increased, overall community evenness remained relatively stable. Boxplot distribution patterns revealed that the Ctrl group had lower and more consistent richness, whereas PD and PD‐RA groups exhibited higher outlier values, indicating greater interindividual variability. The RA group's indices were intermediate between the Ctrl and PD‐RA groups. In summary, the oral microbiota from patients with PD and PD‐RA demonstrated increased richness, with microbial diversity further elevated and more variable in patients with RA and concurrent PD.

The β‐diversity principal coordinates analysis (PCoA) based on unweighted UniFrac distances demonstrated clear separation between the Ctrl and RA groups along the principal axes, with minimal sample overlap. This finding indicates a significant structural difference in oral microbiota composition between healthy controls and patients with RA. Samples from the PD‐RA group were primarily distributed near those of the RA group, with extensive overlap in their 95% confidence ellipses, indicating high similarity in oral microbial composition between patients with RA with and without periodontitis. However, the PD and PD‐RA groups were distinctly separated on the PCoA plot, with virtually no overlap between their ellipses. This finding suggests that the presence of RA significantly alters the oral microbiota of patients with PD, aligning it more closely with that of patients with RA. The extent of ellipse overlap reflects microbial similarity—greater overlap indicates similar community structures, while minimal overlap denotes distinct differences. In this study, considerable overlap between the RA and PD‐RA groups highlights their microbiota resemblance. In contrast, the clear separation between PD and PD‐RA underscores the divergence introduced by RA.

Further LEfSe analysis identified characteristic bacterial genera differentiating the groups. Compared to the PD‐RA group, the RA group showed higher levels of several common oral commensals, including *Selenomonas* sp., *Rothia* sp., *Treponema* sp., *Haemophilus* sp., and *Streptococcus* sp. Conversely, the PD‐RA group was enriched with numerous PD‐associated anaerobic Gram‐negative pathogens such as *Aggregatibacter* sp., *Neisseria* sp., *Actinomyces* sp., *Fusobacterium* sp., *Peptostreptococcus* sp., and *Veillonella* sp., along with key “red complex” bacteria including *Porphyromonas* sp. and *Prevotella* sp. Notably, most genera elevated in the PD‐RA group are typical of periodontal lesions. *P. gingivalis* and *Prevotella* sp., for instance, were scarcely detected in healthy and RA groups, but substantially enriched in the PD‐related samples. This finding suggests that once patients with RA develop PD, their oral microbiota shifts toward a pathogenic profile, with commensals replaced by disease‐associated anaerobes. While dysbiosis is evident in both PD and RA, the specific microbial alterations differ. Saliva from the RA + PD group appears to harbor detrimental microbial traits from both diseases, potentially with a higher pathogen load. These findings support a microbial ecological link between PD and RA.

### 3.2 Metabolomic Characteristics of Patients With Concurrent PD and RA

**FIGURE 2:**
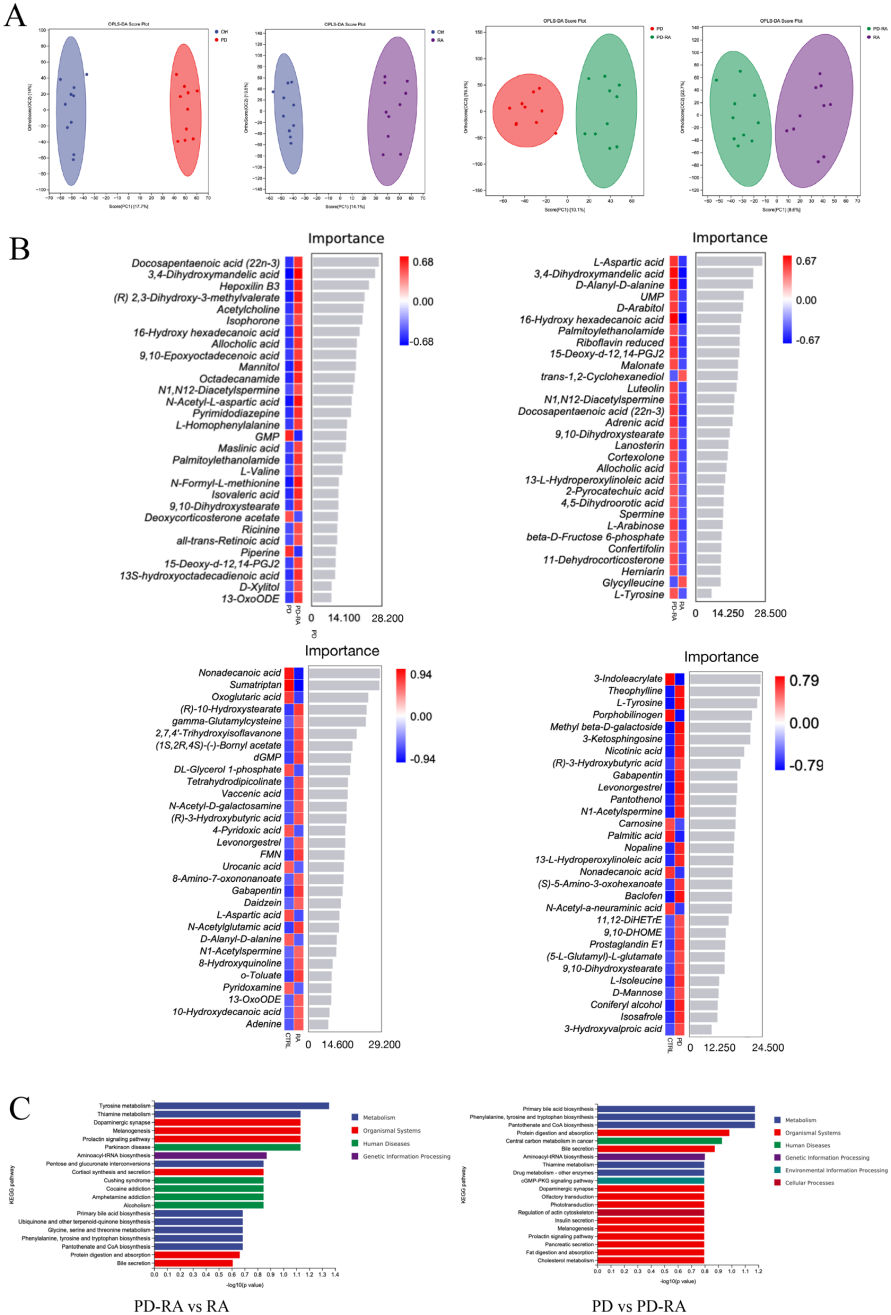
Differences in salivary metabolomic profiles of study subjects. (A) OPLS‐DA score plot comparing metabolic profiles of healthy controls, periodontitis patients, RA patients, and RA patients with periodontitis, demonstrating clear separation between groups. (B) Random forest plot of the most important differential metabolites. (C) KEGG pathway enrichment analysis of differential metabolites. The plot displays pathways that are significantly enriched (sorted by *p*‐value in ascending order); the *x*‐axis represents the degree of enrichment (rich factor) of differential metabolites in each pathway, and the *y*‐axis lists the pathway names. In the OPLS‐DA plot, each point represents a subject sample (different colors/shapes correspond to different groups). In the correlation heatmap, red indicates a positive correlation and blue indicates a negative correlation.

Untargeted metabolomic profiling was performed on saliva samples from the study groups. Orthogonal partial least squares discriminant analysis (OPLS‐DA) revealed distinct metabolic signatures among the groups (Figure 2A). In the OPLS‐DA score plot, both the PD and RA groups were separated from the healthy controls. Notably, the PD‐RA group was further distinguished along the first principal component, indicating a significantly different metabolic profile. The 95% confidence ellipses for each group showed no overlap, suggesting robust between‐group differences.

Differential metabolite analysis was conducted across key group comparisons. Compared to healthy controls, the RA group had 46 significantly altered metabolites (Variable Importance in Projection [VIP] > 1, *p* < 0.05). Elevated in RA were inflammation‐related lipid and amino acid metabolites, such as γ‐glutamylcysteine, 3‐hydroxybutyrate, and 13‐oxo‐octadecadienoic acid (13‐oxoODE). Metabolites that were higher in healthy controls (and decreased in RA) included L‐aspartic acid, D‐alanyl‐D‐alanine, 4‐pyridoxic acid, and pyridoxamine (the latter two being vitamin B_6_ metabolites). These results suggest that patients with RA exhibit disordered amino acid metabolism and increased inflammatory mediators, along with diminished vitamin B₆ derivatives, potentially reflecting the impact of chronic systemic inflammation on host metabolism.

A total of 28 metabolites significantly differed between the PD and PD‐RA groups. The PD group exhibited significantly higher levels of only three metabolites: guanosine monophosphate (GMP), deoxycorticosterone acetate, and piperine, indicating limited metabolic elevation compared to RA + PD. Conversely, the PD‐RA group showed significantly increased levels of numerous metabolites, particularly those involved in lipid metabolism and microbial processes. Notably elevated metabolites in PD‐RA group included docosapentaenoic acid (DPA, 22:5 n‐3, an ω‐3 polyunsaturated fatty acid [PUFA]), 3,4‐dihydroxy mandelic acid, Hepoxilin B3 (a 12‐LOX pathway lipoxin), (R)‐2,3‐dihydroxy‐3‐methylpentanoic acid (a branched‐chain amino acid intermediate), allocholic acid (a secondary bile acid), 9,10‐epoxy octadecenoic acid (a ω‐6 metabolite), mannitol, palmitoylethanolamide (PEA, an endocannabinoid receptor agonist), L‐valine, 9,10‐dihydroxy stearic acid, 15‐deoxy‐Δ^12^,^14^‐prostaglandin J₂ (15d‐PGJ₂, an anti‐inflammatory mediator from arachidonic acid metabolism), 13S‐hydroxy octadecadienoic acid (13S‐HODE), among others. These findings indicate that the PD‐RA group displayed broad metabolic elevation spanning lipids, amino acids, nucleotides, and microbially derived compounds.

Similarly, the comparison of RA versus PD‐RA groups revealed distinct metabolite profiles, partially overlapping with the PD versus PD‐RA comparison. The overall trend is that the PD‐RA group underwent more extensive metabolic reprogramming than either disease alone. Especially prominent were elevated metabolites from arachidonic acid pathways—such as epoxy‐octadecenoic acid and prostaglandin J_2_—as well as disruptions in bile acid metabolism, short‐ and branched‐chain fatty acids, and polyamines. These changes reflect complex dysregulation of both microbial functions and host immunometabolism. For instance, heightened PUFA metabolism may signal upregulated inflammatory mediator synthesis, while alter bile acids and reduced short‐chain fatty acids (SCFAs) suggest compromised gut barrier integrity and metabolic function. The increased levels of anti‐inflammatory molecules like 15d‐PGJ_2_ and PEA in the PD‐RA group may represent compensatory responses to chronic inflammation, though insufficient to counteract the overall pro‐inflammatory milieu.

In summary, the salivary metabolome of patients with coexisting PD and RA revealed a distinct signature of heightened inflammation and microbial dysbiosis. This profile was characterized by the significant upregulation of the arachidonic acid pathway, indicating a pro‐inflammatory lipid mediator storm, as well as an accumulation of microbially‐derived metabolites like secondary bile acids and polyamines, reflecting altered gut microbial activity. Additionally, disturbances in host amino acid and vitamin metabolism suggested systemic metabolic stress. Together, these changes paint a picture of a complex, multisystem metabolic disruption driven by the interplay between the two diseases.

### 3.3 Periodontitis‐Associated Salivary Microbiota Exacerbates Arthritis in CIA Mice

**FIGURE 3:**
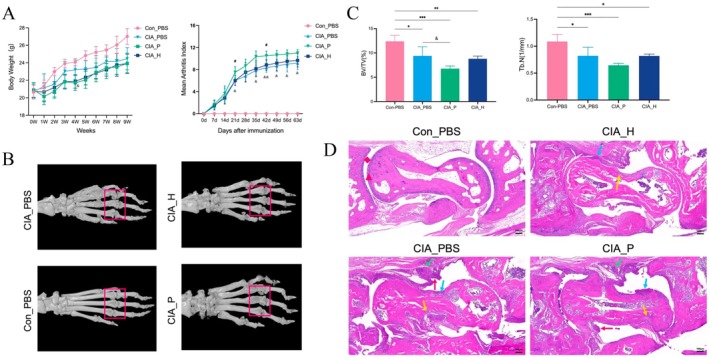
Exacerbation of arthritis phenotype in CIA mice by periodontitis salivary microbiota. (A) Time‐course of arthritis clinical scores in CIA_H (healthy microbiota) versus CIA_P (periodontitis microbiota) mice. The curve shows earlier onset and higher peak arthritis scores in CIA_P mice. (B) 3D micro‐CT reconstructions of mouse hind paws, comparing bone erosion in CIA_H versus CIA_P groups (red boxes highlight erosion sites). The quantitative bar graphs show differences in bone volume fraction (BV/TV) and trabecular number (Tb.N) between the two groups. (D) H&E‐stained sections of ankle joints (Rhombus: Joint space; triangle: Articular cartilage; green arrow: Inflammatory cell infiltration; yellow arrow: Cartilage damage; blue arrow: Synovial hyperplasia; red arrow: Pannus formation).

To assess whether PD‐derived salivary microbiota aggravates RA, in vivo experiments were conducted using the CIA mouse model. The CIA model was considered successfully established based on both clinical and pathological criteria. Clinically, establishment was confirmed when a mouse exhibited a clinical score of ≥ 1 for any paw in two consecutive assessments, or when ankle/dorsum thickness increased by ≥ 15% from baseline with visible erythema and edema. Postmortem radiological and histological confirmation through Micro‐CT scans and H&E staining of joint tissues verified that all mice in the CIA model group developed typical pathological changes of arthritis, including bone erosion, synovial hyperplasia, and pannus formation. CIA mice inoculated with salivary microbiota from patients with PD (CIA_P group) developed considerably more severe arthritis than those receiving microbiota from healthy controls (CIA_H group). By day 24 postimmunization, CIA_P mice exhibited hind paw redness and swelling approximately 3 days earlier than CIA_H mice. Thereafter, arthritis scores in CIA_P mice escalated rapidly, reaching an average of 3.5 ± 0.5 by day 42, significantly higher than the CIA_H group (2.3 ± 0.4, *p* < 0.05) (Figure 3A). By day 63, four CIA_P mice developed joint ankylosis, and cumulative arthritis scores remained significantly elevated compared to the CIA_H group (*p* < 0.05). As an additional control, the CIA_phosphate buffered saline (PBS) group (mice gavaged with PBS alone) showed arthritis scores comparable to the CIA_H group, indicating that PBS gavage had no significant impact on disease progression. However, the normal control group (Con_PBS) exhibited no signs of arthritis throughout the study.

Micro‐computed tomography (CT) imaging confirmed that PD‐associated microbiota aggravated joint bone destruction (Figure 3B). Three‐dimensional reconstructions of the hind paw joints revealed more extensive bone erosions in CIA_P mice (highlighted by red boxes) compared to CIA_H mice. Quantitative analysis showed a decreased bone volume fraction (bone volume [BV]/ total volume [TV]) in CIA_P mice, indicating greater bone loss. Histopathological examination of ankle joint sections further revealed more pronounced synovial hyperplasia and inflammatory cell infiltration in the CIA_P group. These findings demonstrate that colonization with PD‐derived oral microbiota significantly worsened joint pathology in CIA mice, both histologically and radiographically.

**FIGURE 4:**
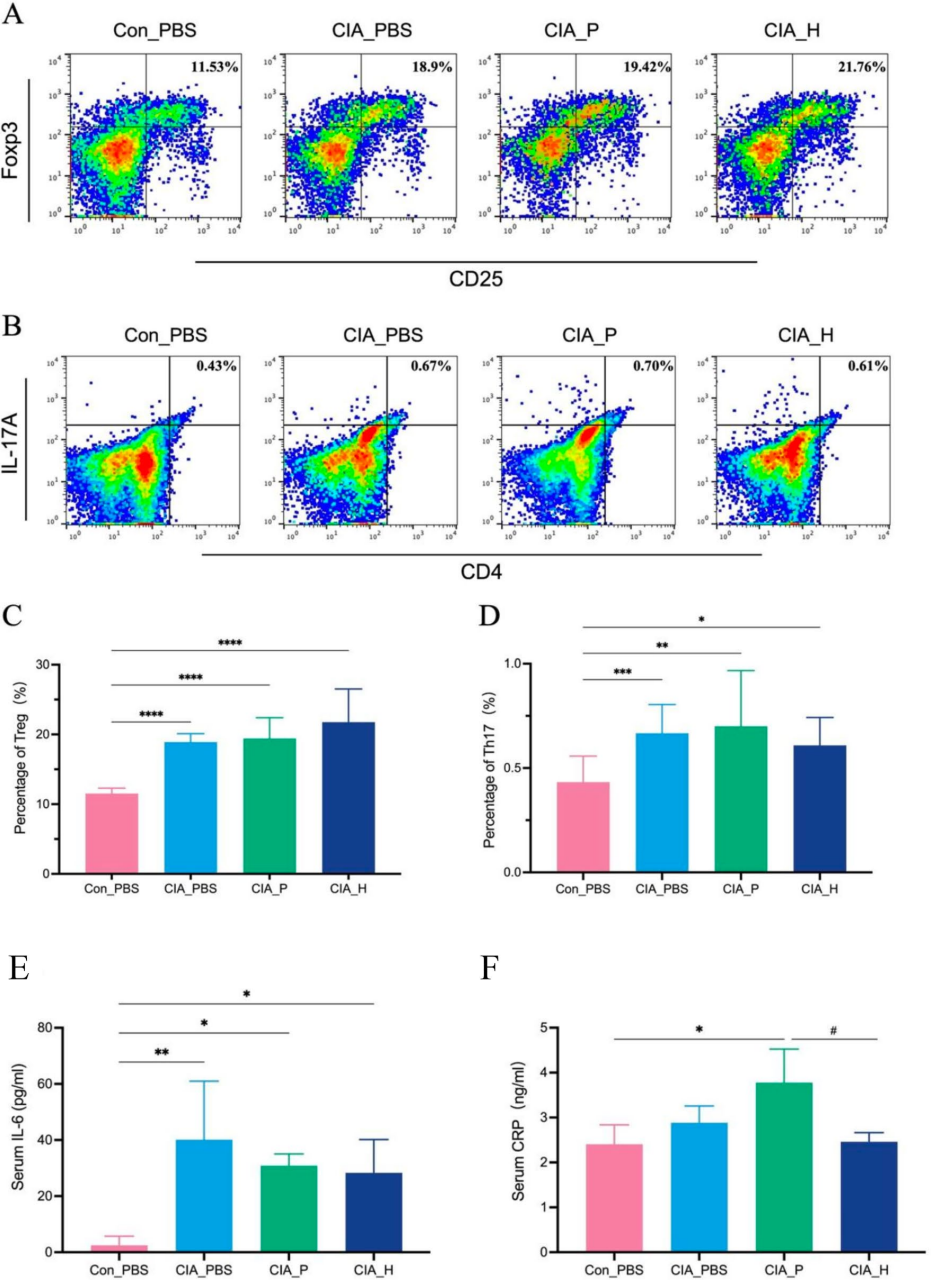
Impact of periodontitis microbiota on inflammatory factors and T cell subsets in CIA mice. (A) Representative flow cytometry dot plot for Treg cells (CD4^+^Foxp3^+^) in mouse spleen. (B) Representative flow cytometry dot plot for Th17 cells (CD4^+^IL‐17A^+^) in mouse spleen. (C) Comparison of Treg cell proportions in spleen (percentage of CD4^+^ T cells). (D) Comparison of Th17 cell proportions in spleen. (*: Compared to Con_PBS group, *p* < 0.05; **: *p* < 0.01; ***: *p* < 0.001; ****: *p* < 0.0001.) (E) Serum IL‐6 levels in mice. (F) Serum CRP levels in mice. (*: Compared to Con_PBS group, *p* < 0.05; **: *p* < 0.01. #: CIA_P group versus CIA_H group, *p* < 0.05).

Regarding systemic inflammation, CIA_P mice exhibited significantly higher serum C‐reactive protein (CRP) levels than CIA_H mice (*p* < 0.05). Interleukin‐6 (IL‐6) levels were also elevated, although insignificantly (Figure 4). As a key pro‐inflammatory cytokine involved in both RA and PD, IL‐6, alongside CRP, a marker of systemic inflammation, suggests that periodontitis microbiota induced a more intense systemic inflammatory response, mirroring clinical findings in patients with RA and concomitant PD. Flow cytometry further supported the presence of immune dysregulation (Figure 4). Analysis of CD4^+^ T cell subsets in the spleens of CIA_P mice revealed a reduction in regulatory T cells (Tregs) and an increase in pro‐inflammatory Th17 cells compared to CIA_H mice. This shift indicates that PD microbiota disrupted T cell balance, promoting a Th17‐dominant pro‐inflammatory response. The observed IL‐6 elevation (known to promote Th17 differentiation and inhibit Tregs) supports the notion that the gut‐colonizing periodontal pathogens triggered IL‐6‐mediated Th17 expansion, intensifying autoimmune responses in CIA mice.

### 3.4 Gut Microbiota Alterations Induced by PD Microbiota

**FIGURE 5:**
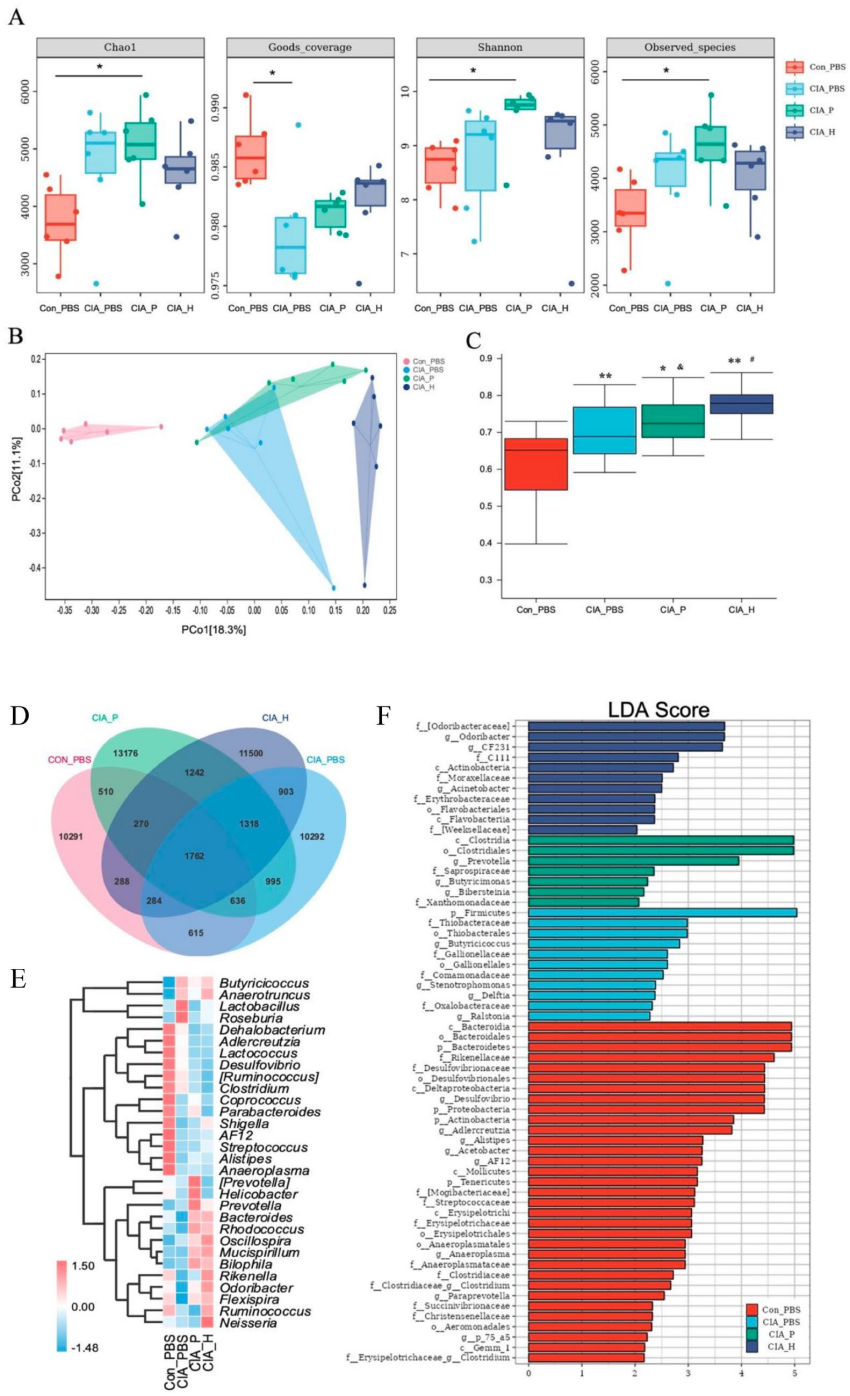
Analysis of cecal microbiota diversity in mice. (A) Alpha diversity analysis of cecal microbiota (Chao1 index, Good's coverage, Shannon index, Observed_species index). In each boxplot, whiskers indicate the minimum and maximum values, and the central line indicates the median (*: *p* < 0.05). (B) Principal coordinates analysis (PCoA) of cecal microbiota beta diversity. (C) Between‐group differential analysis of microbial communities (*: compared to Con_PBS group, *p* < 0.05; **: *p* < 0.01. &: CIA_P group versus CIA_PBS group, *p* < 0.05; #: CIA_P group versus CIA_H group, *p* < 0.05). (D) Venn diagram showing the number of shared and unique ASVs/OTUs in each group's cecal microbiota. (E) Heatmap of the relative abundance of the top 30 genera in cecal microbiota across the four groups. (F) LEfSe analysis of cecal microbiota; only taxa with LDA score ≥ 2 are shown.

To investigate colonization and its effects on gut ecology, the cecal contents from CIA mice were analyzed 6 weeks after gavage using 16S rRNA sequencing (Figure 5). Compared to the CIA_H group, the CIA_P mice exhibited a modest decline in alpha diversity and showed some differences in beta diversity, although considerable intragroup variability was observed. This observation indicates variable colonization efficacy of the transplanted saliva microbiota among individual mice. Despite this, Linear Discriminant Analysis Effect Size (LEfSe) analysis identified several gut taxa enriched in the CIA_P group, particularly members of the Prevotellaceae and Bacteroidaceae families; Lactobacillaceae was more abundant in the CIA_H group. These findings suggest that anaerobic bacteria from PD saliva (such as *Prevotella* spp.) were able to survive in the gut and partially reshape its microbial profile, introducing PD‐associated features. Conversely, healthy saliva microbiota, rich in beneficial genera such as *Lactobacillus* and *Rothia*, likely supported a more balanced gut ecosystem.

**FIGURE 6:**
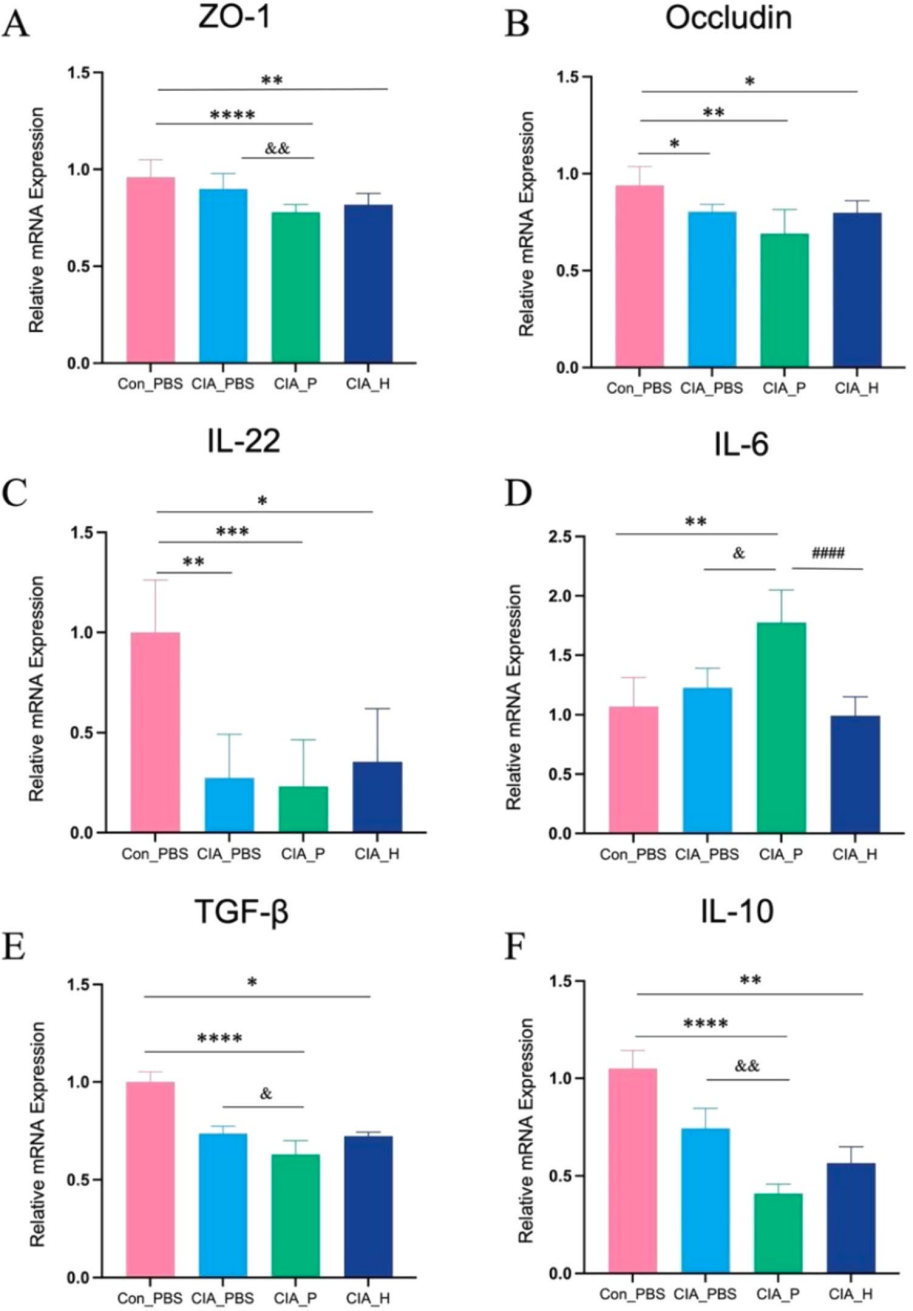
Colonic tissue and mesenteric lymph node mRNA expression levels of barrier and immune regulators. (A) ZO‐1; (B) Occludin; (C) IL‐22; (D) IL‐6; (E) TGF‐β; (F) IL‐10. (*: Compared to Con_PBS group, *p* < 0.05; **: *p* < 0.01; ***: *p* < 0.001; ****: *p* < 0.0001. &: CIA_P group versus CIA_PBS group, *p* < 0.05; &&: *p* < 0.01. #: CIA_P group versus CIA_H group, ####: *p* < 0.0001).

The qRT‐PCR analysis revealed reduced expression of tight junction proteins zonula occludens‐1 (ZO‐1) and occludin in the colonic mucosa of CIA mice (Figure 6). Notably, colonic ZO‐1 expression in the CIA_P group was significantly lower than in the CIA_PBS group (*p* < 0.05), indicating that colonization with PD microbiota compromised the gut barrier integrity and increased intestinal permeability. Correspondingly, IL‐22 expression in colonic tissue was substantially downregulated, while IL‐6 expression was upregulated in CIA_P mice (*p* < 0.05) (Figure 6). Furthermore, mesenteric lymph node analysis showed considerably reduced expression of anti‐inflammatory cytokines IL‐10 and transforming growth factor‐β (TGF‐β) in the CIA_P group compared to both the CIA_PBS and CIA_H groups (*p* < 0.05) (Figure 6) reflecting disrupted immune homeostasis induced by PD‐associated microbiota.

## 4 Discussion

This study, integrating clinical cohort analysis with animal experiments, systematically revealed the “microbiota‐metabolism‐immunity” axis underlying the interaction between PD and RA. The main findings include:

Finding 1: Patients with PD and RA exhibited increased oral microbial richness and diversity, marked by enrichment of classical periodontal pathogens (such as *Porphyromonas* sp. and *Prevotella* sp.), along with significant alterations in systemic metabolic profiles, particularly in lipid and microbe‐associated metabolites.

Finding 2: Salivary microbiota from patients with PD successfully colonized the gut of arthritic mice, altering the microbial composition, downregulating barrier‐related proteins (such as ZO‐1 and occludin), and suppressing anti‐inflammatory cytokines (such as IL‐10 and TGF‐β). These disruptions impaired intestinal immune homeostasis, skewed immunity toward Th17‐driven inflammation, and exacerbated both arthritis severity and joint pathology.

First, a strong microbiome‐level link was established between PD and RA. Our findings show that anaerobic genera such as *Porphyromonas* sp., *Prevotella* sp., *Tannerella* sp., *Treponema* sp., *Fusobacterium* sp., and *Aggregatibacter* sp., coenriched in patients with PD and RA, contribute to local inflammation via mechanisms including peptidylarginine deiminase (PPAD)‐mediated protein citrullination, leukotoxin A (LtxA)‐induced excessive neutrophil extracellular trap (NET)osis, and Fusobacterium adhesin A (FadA)‐triggered mucosal disruption. These pathogens may disseminate via the bloodstream or through swallowing, compromising epithelial barriers, disrupting receptor activator of nuclear factor κB‐ligand (RANKL) regulation, and promoting osteoclastogenesis. The downstream effectors—anti‐citrullinated protein antibody (ACPA), IL‐17, and TNF‐α—drive a shared inflammatory and osteolytic process in synovial and periodontal tissues. Increased susceptibility to PD in patients with RA and differences in their oral microbiota compared to healthy controls have been reported [8, 14, 18, 19, 20]. Our data refine this understanding: patients with RA harbor detectable levels of periodontal pathogens even when periodontal indices are clinically normal, implying that RA's systemic inflammation induces subclinical oral dysbiosis. Anaerobes typically confined to periodontal pockets may persist in saliva. With overt PD, their abundance increases further. This phenomenon suggests a reciprocal microbial‐ecological mechanism: RA‐related immune dysfunction impairs oral mucosal defenses, facilitating pathogen colonization; in turn, these microbes aggravate local inflammation and propagate systemic inflammatory signals [21, 22]. Notably, significant quantities of *Actinomyces* sp., *Peptostreptococcus* sp., and other facultative anaerobes—organisms also commonly present in the gut—were identified in the saliva of patients with PD and RA. When swallowed in large numbers, these bacteria can disrupt the gut microbial balance. Of particular concern is *Prevotella* sp., previously shown to expand abnormally in the gut of patients with early RA. The expansion of *Prevotella* in the gut can activate mucosal immune cells via pattern recognition receptors, triggering the release of pro‐inflammatory cytokines such as IL‐1β, IL‐6, and IL‐23. These cytokines drive the differentiation of naïve CD4^+^ T cells into pathogenic Th17 cells. Subsequently, these gut‐derived Th17 cells can migrate to the joints, where they promote osteoclastogenesis and synovial inflammation, thus establishing a key link between gut dysbiosis and arthritis pathology [23, 24]. Our results indicate that the oral cavity, especially in PD, may serve as a persistent reservoir of *Prevotella* sp., thereby promoting RA pathogenesis.

Second, metabolomic profiling revealed significant metabolic remodeling in patients with RA and coexisting periodontitis, distinct from either condition alone. These patients exhibited widespread accumulation of metabolites linked to inflammation and microbial activity. A key finding was the altered metabolism of unsaturated fatty acids, particularly elevated arachidonic acid derivatives. These mediators, including prostaglandins and leukotrienes, are central to tissue destruction in both RA and PD, contributing to bone resorption and soft tissue inflammation [25, 26, 27]. Therefore, PD might amplify RA‐associated inflammation by expanding the host's arachidonic acid pool and its downstream mediators. This hypothesis aligns with prior studies: in a DSS‐induced colitis model, transplantation of salivary microbiota from periodontitis‐affected mice enhanced arachidonic acid metabolism, reduced unsaturated fatty acids, and exacerbated colonic inflammation [12]. Moreover, our metabolic data revealed an accumulation of microbe‐derived metabolites in patients with coexisting RA and PD. N1, N12‐diacetylspermine, a polyamine produced by gut microbiota and linked to inflammatory milieu in conditions such as colorectal cancer [28], was significantly elevated in patients with RA + PD, possibly indicating altered gut microbial composition and metabolism. These microbial metabolites may either directly influence host immune cells or act as danger signals by engaging pattern recognition receptors, thereby amplifying systemic inflammation. In summary, the coexistence of RA and PD induces a heightened inflammation‐driven metabolic state, potentially facilitating mutual disease exacerbation.

Our animal experiments further demonstrated that PD‐associated oral microbiota promotes RA progression. Unlike previous studies that focused on individual pathogens (such as *P. gingivalis* ), a clinically relevant approach was adopted by transplanting the entire salivary microbiota. This experiment allowed a comprehensive assessment of how the PD‐associated microbial community influences host immunity. The gavage of arthritic mice with microbiota from patients with PD significantly aggravated clinical and pathological features compared to microbiota from healthy donors. Colonization by these microbes impaired gut barrier function and disrupted mucosal immune homeostasis, thereby intensifying systemic inflammation and joint pathology. Mice in the CIA_P group exhibited reduced expression of ZO‐1 and occludin in the colon, indicating compromised intestinal tight junctions and increased permeability. This epithelial disruption facilitates the translocation of microbial products into circulation, triggering systemic inflammation [29]. Moreover, IL‐22 levels in the colonic mucosa were substantially decreased. As a key cytokine for epithelial integrity, IL‐22 promotes mucosal defense and limits microbial translocation [30]. Its reduction further impairs barrier repair and antimicrobial responses, enabling persistent inflammation. Conversely, local IL‐6 levels were substantially elevated in CIA_P mice, reflecting a pro‐inflammatory milieu. IL‐6 promotes Th17 differentiation, especially when coupled with TGF‐β, driving naive T cells toward a Th17 phenotype that produces IL‐17 and IL‐22 [31]. Furthermore, IL‐10 and TGF‐β levels in the mesenteric lymph nodes were considerably lower in CIA_P mice than in CIA_PBS or CIA_H controls, suggesting impaired Treg‐mediated immunoregulation. These cytokines are essential for immune tolerance and the suppression of inflammation [32, 33]. Their downregulation indicates a shift away from anti‐inflammatory responses toward unchecked inflammation. Collectively, these changes reveal a Th17/Treg imbalance in CIA_P mice, characterized by elevated Th17 activity and insufficient Treg function, consistent with our observed increase in the Th17/Treg ratio.

The choice to use mixed salivary microbiota rather than a single bacterial strain was deliberate and grounded in translational rationale. Previous seminal studies have shown that *P. gingivalis* alone can aggravate arthritis through immune and microbiota‐mediated mechanisms. For example, Sato et al. demonstrated that oral administration of *P. gingivalis* exacerbated collagen‐induced arthritis (CIA) by disrupting gut microbial composition, expanding Th17 cells, and worsening joint destruction [34]. Similarly, Maresz et al. found that *P. gingivalis* infection accelerated the onset and increased the severity of arthritis in mice through peptidylarginine deiminase–mediated protein citrullination and enhanced autoimmune responses [35]. These findings have established a clear mechanistic link between periodontal pathogens and RA exacerbation. However, periodontitis is not driven by a single pathogen but by a polymicrobial dysbiosis, in which keystone species interact synergistically with commensals to alter the host's immune tone and metabolic environment. Therefore, using the entire salivary microbiota more accurately replicates the ecological complexity and collective pathogenic potential of clinical periodontitis. This community‐based transplantation model offers several advantages. It reproduces the multispecies interactions, metabolic cross‐feeding, and signaling networks that underlie oral dysbiosis and allows the integration of microbiome, metabolome, and immune data to elucidate systemic mechanisms along the oral–gut–joint axis. In contrast, single‐strain infection models, though valuable for mechanistic dissection, cannot capture the community‐level dynamics or the metabolic and immunological heterogeneity inherent in the disease. By incorporating both healthy and PBS controls, our study design effectively distinguishes the effects of periodontitis‐associated dysbiosis from those of healthy oral microbiota and procedural factors, thereby ensuring robust and physiologically relevant conclusions.

Based on these results, it is proposed that PD‐related microbiota exacerbate RA by first colonizing the gut, displacing beneficial commensals such as *Lactobacillus* sp. and *Rothia* sp., potentially reducing short‐chain‐fatty‐acid (SCFA) producers, and fostering a pro‐inflammatory environment enriched in protein‐fermentation products like isovalerate. This dysbiosis, dominated by Gram‐negative pathogens, releases outer‐membrane vesicles and endotoxins that disrupt tight junctions, increase intestinal permeability, and permit bacterial toxins and metabolites to enter the circulation. These microbial components activate mucosal and mesenteric lymph node immune cells, expand pro‐inflammatory Th17 cells, suppress Tregs, and polarize macrophages toward an M1 phenotype that secretes IL‐6, IL‐1β, and TNF—amplifying synovial inflammation. Consequently, systemic immune activation triggered by gut barrier compromise, in conjunction with genetic or immune predisposition, intensifies autoimmune joint responses. This model illustrates how microbial dysbiosis and host susceptibility interact to drive RA progression.

Our study revealed several noteworthy findings. First, patients with both PD and RA exhibited elevated salivary levels of anti‐inflammatory mediators such as 15d‐PGJ_2_ and palmitoylethanolamide (PEA). These molecules are well‐ recognized as endogenous regulators that suppress inflammation and promote resolution [36, 37, 38]. Their elevation in such a highly pro‐inflammatory setting likely reflects a compensatory by the body. However, under the persistent and severe inflammation associated with coexisting PD and RA, this response is insufficient, indicating a dysregulated immune state in which the body is unable to restore balance.

In summary, our study reveals a novel mechanism through which PD and RA aggravate each other through an “oral‐gut axis.” Dysbiotic salivary microbiota in PD can disrupt gut microbial and metabolic homeostasis, trigger systemic inflammation and immune imbalance, and exacerbate tissue destruction in joints and periodontal structures in RA‐susceptible hosts. In our animal experiment, we utilized a single donor for each microbiota transplantation group. While this approach was effective for providing a strong proof and demonstrating the pathogenic potential of PD‐associated microbiota, the results may be influenced by donor‐specific characteristics. Future studies could strengthen the generalizability of these results by using pooled microbiota from multiple donors or validating findings with additional individual samples. Moreover, our findings are significant as they successfully establish a mechanistic link via the oral‐gut axis and lay the essential groundwork for future, larger‐scale investigations. Furthermore, our conclusion regarding the reduction of SCFAs in the animal model is based on indirect evidence, namely, clinical metabolomic results and changes in SCFA‐producing bacteria, rather than direct quantification in mice. This mechanistic inference, while plausible, requires future validation through targeted metabolomic analyses. Taken together, these findings highlight the systemic impact of oral health on autoimmune diseases. For RA management, proactive prevention and treatment of oral infections may be crucial, while in PD patients, early control of systemic inflammation and monitoring of rheumatologic markers may help reduce the risk of RA or related disorders. Our results provide a theoretical foundation for therapeutic strategies that target both conditions simultaneously. Integrated approaches, such as rigorous oral hygiene and modulation of gut microbiota, may help disrupt this pathological feedback loop and improve patient outcomes.

## Author Contributions

Ruiyang Ge contributed to data acquisition, analysis, and interpretation, drafted and critically revised the manuscript. Rong Liu contributed to data acquisition, analysis, and interpretation. Yingying Zhou, Ziyao Zhuang, Haowei Mao, Wenzheng Liao, Lei Han contributed to data acquisition. Di Cui, Wenrong Yang contributed to conception, design and critically revised the manuscript. Fuhua Yan contributed to conception, design, and data interpretation, critically revised the manuscript. All authors read and approved the final manuscript as submitted.

## Funding

This study was supported by the grants from the National Natural Science Foundation of China (No. 82301101); the grants from the Natural Science Foundation of Jiangsu Province (BK20230159); Lhasa Key Science and Technology Project (Jiangsu Aid Tibet) (No. LSKJ202434); Jiangsu Provincial Medical Key Discipline Cultivation Unit (No. JSDW202246); High‐Level Hospital Construction Project of Nanjing Stomatological Hospital, Affiliated Hospital of Medical School, Institute of Stomatology, Nanjing University (No. 0224C001); Nanjing Medical Science and Technique Development Foundation (No. YKK22179).

## Ethics Statement

The study was conducted in accordance with the Declaration of Helsinki, and approved by the Ethics Committee of the Affiliated Hospital of Medical School, Institute of Stomatology, Nanjing University (approval number: NJSH‐2022NL‐059).

## Consent

Informed consent was obtained from all subjects involved in the study.

## Conflicts of Interest

The authors declare no conflicts of interest.

## Supporting information


**FIGURE S1:** fsb271282‐sup‐0001‐FigureS1.pdf.

## Data Availability

All raw data will be made available upon request.

## References

[1] G. Hajishengallis , “Interconnection of Periodontal Disease and Comorbidities: Evidence, Mechanisms, and Implications,” Periodontology 2000 89, no. 1 (2022): 9–18. 35244969 10.1111/prd.12430 PMC9018559

[2] I. B. Mcinnes and G. Schett , “The Pathogenesis of Rheumatoid Arthritis,” New England Journal of Medicine 365, no. 23 (2011): 2205–2219. 22150039 10.1056/NEJMra1004965

[3] A. Di Matteo , J. M. Bathon , and P. Emery , “Rheumatoid Arthritis,” Lancet 402, no. 10416 (2023): 2019–2033. 38240831 10.1016/S0140-6736(23)01525-8

[4] P. Brown , A. G. Pratt , and K. L. Hyrich , “Therapeutic Advances in Rheumatoid Arthritis,” BMJ (Clinical Research Ed.) 384 (2024): e070856. 10.1136/bmj-2022-070856 38233032

[5] J. Schmickler , A. Rupprecht , S. Patschan , et al., “Cross‐Sectional Evaluation of Periodontal Status and Microbiologic and Rheumatoid Parameters in a Large Cohort of Patients With Rheumatoid Arthritis,” Journal of Periodontology 88, no. 4 (2017): 368–379. 27858553 10.1902/jop.2016.160355

[6] M. De Smit , J. Westra , A. Vissink , B. Doornbos‐van der Meer , E. Brouwer , and A. J. van Winkelhoff , “Periodontitis in Established Rheumatoid Arthritis Patients: A Cross‐Sectional Clinical, Microbiological and Serological Study,” Arthritis Research & Therapy 14, no. 5 (2012): R222. 23075462 10.1186/ar4061 PMC3580533

[7] L. Massarenti , C. Enevold , D. Damgaard , et al., “Peptidylarginine Deiminase 2 Gene Polymorphisms in Subjects With Periodontitis Predispose to Rheumatoid Arthritis,” International Journal of Molecular Sciences 23, no. 17 (2022): 9536. 36076933 10.3390/ijms23179536 PMC9455246

[8] A. I. Bolstad , B. S. Fevang , and S. A. Lie , “Increased Risk of Periodontitis in Patients With Rheumatoid Arthritis: A Nationwide Register Study in Norway,” Journal of Clinical Periodontology 50, no. 8 (2023): 1022–1032. 37202856 10.1111/jcpe.13826

[9] Y. Qiao , Z. Wang , Y. Li , Y. Han , Y. Zhou , and X. Cao , “Rheumatoid Arthritis Risk in Periodontitis Patients: A Systematic Review and Meta‐Analysis,” Joint Bone Spine 87, no. 6 (2020): 556–564. 32593704 10.1016/j.jbspin.2020.04.024

[10] S.‐Y. Park , B.‐O. Hwang , M. Lim , et al., “Oral–Gut Microbiome Axis in Gastrointestinal Disease and Cancer,” Cancers 13, no. 9 (2021): 2124. 33924899 10.3390/cancers13092124 PMC8125773

[11] J. Bao , L. Li , Y. Zhang , et al., “Periodontitis May Induce Gut Microbiota Dysbiosis via Salivary Microbiota,” International Journal of Oral Science 14, no. 1 (2022): 32. 35732628 10.1038/s41368-022-00183-3 PMC9217941

[12] J. Qian , J. Lu , Y. Huang , et al., “Periodontitis Salivary Microbiota Worsens Colitis[J],” Journal of Dental Research 100, no. 12 (2021): 1340–1350. 10.1177/00220345211049781 34796773

[13] T. Kobayashi and P. M. Bartold , “Periodontitis and Periodontopathic Bacteria as Risk Factors for Rheumatoid Arthritis: A Review of the Last 10 Years,” Japanese Dental Science Review 59 (2023): 263–272. 37674898 10.1016/j.jdsr.2023.08.002 PMC10477376

[14] J. U. Scher , C. Ubeda , M. Equinda , et al., “Periodontal Disease and the Oral Microbiota in New‐Onset Rheumatoid Arthritis,” Arthritis and Rheumatism 64, no. 10 (2012): 3083–3094. 22576262 10.1002/art.34539 PMC3428472

[15] Y. Feng , Z. Chen , S. Tu , et al., “Role of Interleukin‐17A in the Pathomechanisms of Periodontitis and Related Systemic Chronic Inflammatory Diseases,” Frontiers in Immunology 13 (2022): 862415. 35371044 10.3389/fimmu.2022.862415 PMC8968732

[16] N. Inanc , G. Mumcu , M. Can , et al., “Elevated Serum TREM‐1 Is Associated With Periodontitis and Disease Activity in Rheumatoid Arthritis,” Scientific Reports 11, no. 1 (2021): 2888. 33536478 10.1038/s41598-021-82335-9 PMC7859204

[17] N. Wegner , R. Wait , A. Sroka , et al., “Peptidylarginine Deiminase From *Porphyromonas Gingivalis* Citrullinates Human Fibrinogen and α‐Enolase: Implications for Autoimmunity in Rheumatoid Arthritis,” Arthritis & Rheumatism 62, no. 9 (2010): 2662–2672. 20506214 10.1002/art.27552 PMC2941529

[18] T. R. Mikuls , J. B. Payne , F. Yu , et al., “Periodontitis and *Porphyromonas Gingivalis* in Patients With Rheumatoid Arthritis,” Arthritis and Rheumatology 66, no. 5 (2014): 1090–1100. 24782175 10.1002/art.38348 PMC4115329

[19] K. Eriksson , G. Fei , A. Lundmark , et al., “Periodontal Health and Oral Microbiota in Patients With Rheumatoid Arthritis,” Journal of Clinical Medicine 8, no. 5 (2019): 630. 31072030 10.3390/jcm8050630 PMC6572048

[20] J. D. Corrêa , G. R. Fernandes , D. C. Calderaro , et al., “Oral Microbial Dysbiosis Linked to Worsened Periodontal Condition in Rheumatoid Arthritis Patients,” Scientific Reports 9 (2019): 8379. 31182740 10.1038/s41598-019-44674-6 PMC6557833

[21] R. C. Brewer , T. V. Lanz , C. R. Hale , et al., “Oral Mucosal Breaks Trigger Anti‐Citrullinated Bacterial and Human Protein Antibody Responses in Rheumatoid Arthritis,” Science Translational Medicine 15, no. 684 (2023): eabq8476. 36812347 10.1126/scitranslmed.abq8476 PMC10496947

[22] M. F. Konig , L. Abusleme , J. Reinholdt , et al., “ *Aggregatibacter Actinomycetemcomitans*‐Induced Hypercitrullination Links Periodontal Infection to Autoimmunity in Rheumatoid Arthritis,” Science Translational Medicine 8, no. 369 (2016): 369ra176. 10.1126/scitranslmed.aaj1921 PMC5384717 27974664

[23] Y. Maeda , T. Kurakawa , E. Umemoto , et al., “Dysbiosis Contributes to Arthritis Development via Activation of Autoreactive T Cells in the Intestine[J],” Arthritis and Rheumatology 68, no. 11 (2016): 2646–2661. 27333153 10.1002/art.39783

[24] Y. Huang , J. Tang , Z. Cai , et al., “Prevotella Induces the Production of Th17 Cells in the Colon of Mice,” Journal of Immunology Research 2020 (2020): 9607328. 33204736 10.1155/2020/9607328 PMC7657696

[25] J. M. McCoy , J. R. Wicks , and L. P. Audoly , “The Role of Prostaglandin E2 Receptors in the Pathogenesis of Rheumatoid Arthritis,” Journal of Clinical Investigation 110, no. 5 (2002): 651–658. 12208866 10.1172/JCI15528 PMC151107

[26] L. Bouchareychas , E. M. Grössinger , M. Kang , H. Qiu , and I. E. Adamopoulos , “Critical Role of LTB_4_/BLT1 in IL‐23‐Induced Synovial Inflammation and Osteoclastogenesis via NF‐κB,” Journal of Immunology (Baltimore, Md.: 1950) 198, no. 1 (2017): 452–460. 27895169 10.4049/jimmunol.1601346 PMC5173389

[27] Y. Zubery , C. R. Dunstan , B. M. Story , et al., “Bone Resorption Caused by Three Periodontal Pathogens In Vivo in Mice Is Mediated in Part by Prostaglandin,” Infection and Immunity 66, no. 9 (1998): 4158–4162. 9712762 10.1128/iai.66.9.4158-4162.1998 PMC108500

[28] C. H. Johnson , C. M. Dejea , D. Edler , et al., “Metabolism Links Bacterial Biofilms and Colon Carcinogenesis,” Cell Metabolism 21, no. 6 (2015): 891–897. 25959674 10.1016/j.cmet.2015.04.011 PMC4456201

[29] I. Robles‐Vera , A. Jarit‐Cabanillas , P. Brandi , et al., “Microbiota Translocation Following Intestinal Barrier Disruption Promotes Mincle‐Mediated Training of Myeloid Progenitors in the Bone Marrow,” Immunity 58, no. 2 (2025): 381–396.e9. 39848243 10.1016/j.immuni.2024.12.012 PMC11832192

[30] A. M. Hammer , N. L. Morris , A. R. Cannon , et al., “Interleukin‐22 Prevents Microbial Dysbiosis and Promotes Intestinal Barrier Regeneration Following Acute Injury,” Shock 48, no. 6 (2017): 657–665. 28498296 10.1097/SHK.0000000000000900 PMC5681896

[31] E. Bettelli , Y. Carrier , W. Gao , et al., “Reciprocal Developmental Pathways for the Generation of Pathogenic Effector TH17 and Regulatory T Cells,” Nature 441, no. 7090 (2006): 235–238. 16648838 10.1038/nature04753

[32] Y. P. Rubtsov , J. P. Rasmussen , E. Y. Chi , et al., “Regulatory T Cell‐Derived Interleukin‐10 Limits Inflammation at Environmental Interfaces,” Immunity 28, no. 4 (2008): 546–558. 18387831 10.1016/j.immuni.2008.02.017

[33] M. O. Li , Y. Y. Wan , and R. A. Flavel , “T Cell‐Produced Transforming Growth Factor‐β1 Controls T Cell Tolerance and Regulates Th1‐ and Th17‐Cell Differentiation[J],” Immunity 26, no. 5 (2007): 579–591. 17481928 10.1016/j.immuni.2007.03.014

[34] K. Sato , N. Takahashi , T. Kato , et al., “Aggravation of Collagen‐Induced Arthritis by Orally Administered *Porphyromonas Gingivalis* Through Modulation of the Gut Microbiota and Gut Immune System,” Scientific Reports 7, no. 1 (2017): 6955. 28761156 10.1038/s41598-017-07196-7 PMC5537233

[35] K. J. Maresz , A. Hellvard , A. Sroka , et al., “ *Porphyromonas gingivalis* Facilitates the Development and Progression of Destructive Arthritis Through Its Unique Bacterial Peptidylarginine Deiminase (PAD),” PLoS Pathogens 9, no. 9 (2013): e1003627. 24068934 10.1371/journal.ppat.1003627 PMC3771902

[36] M. Ricote , A. C. Li , T. M. Willson , C. J. Kelly , and C. K. Glass , “The Peroxisome Proliferator‐Activated Receptor‐γ Is a Negative Regulator of Macrophage Activation,” Nature 391, no. 6662 (1998): 79–82. 9422508 10.1038/34178

[37] F. Borrelli , B. Romano , S. Petrosino , et al., “Palmitoylethanolamide, a Naturally Occurring Lipid, Is an Orally Effective Intestinal Anti‐Inflammatory Agent,” British Journal of Pharmacology 172, no. 1 (2015): 142–158. 25205418 10.1111/bph.12907 PMC4280974

[38] A. Calignano , G. La Rana , A. Giuffrida , and D. Piomelli , “Control of Pain Initiation by Endogenous Cannabinoids,” Nature 394, no. 6690 (1998): 277–281. 9685157 10.1038/28393

