# Supplementary material for: Periodontitis Salivary Microbiota Exacerbates Murine Rheumatoid Arthritis via Gut Dysbiosis and Immune Dysregulation
Source: FASEB J. 2025 Dec 1;39(23):e71282. doi: 10.1096/fj.202502610R (PMC12666613; doi:10.1096/fj.202502610R)
Supplement: Supplementary file 1 — FIGURE S1: fsb271282‐sup‐0001‐FigureS1.pdf. [file FSB2-39-e71282-s001.pdf]

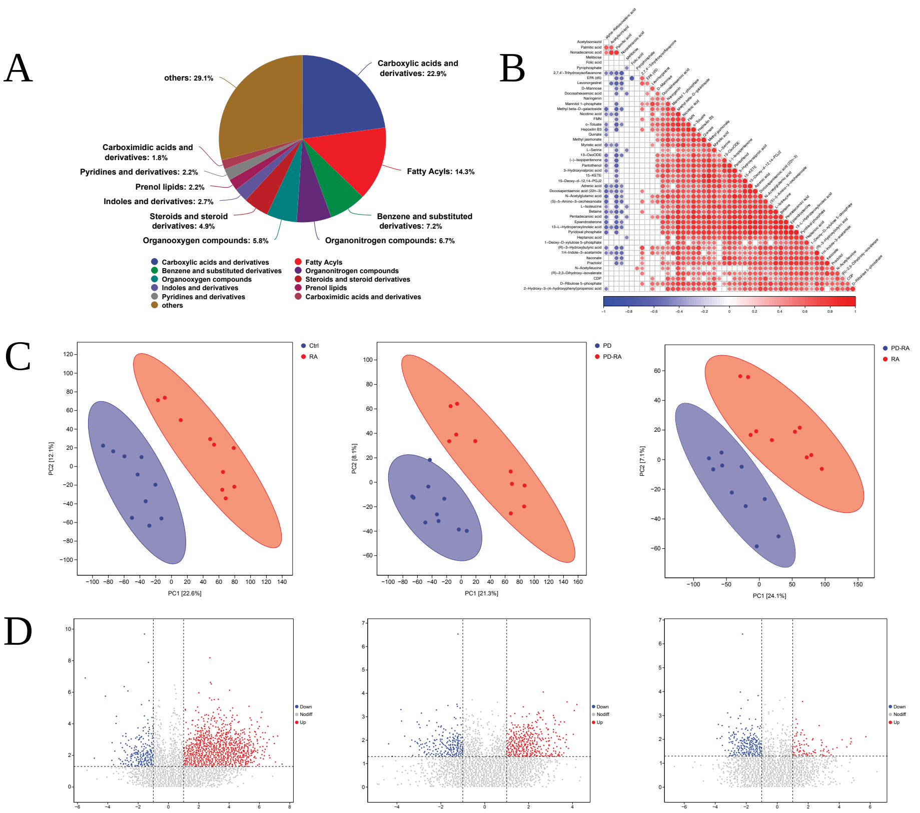

Figure S1. Differences in salivary metabolomic profiles of study subjects. (A) Metabolite identification and classification. Each colored tile denotes a chemical class; the percentage indicates the fraction of identified metabolites assigned to that class. The top 10 classes are shown. (B) Correlation analysis of differential metabolites. Both axes list the names of differential metabolites. The color scale represents the correlation coefficient (red, positive; blue, negative); darker colors indicate stronger correlations. (C) PLS-DA score plot comparing metabolic profiles of healthy controls, periodontitis patients, and RA patients with periodontitis, demonstrating clear separation between groups. (D) Univariate analysis (volcano plot). The x-axis shows log2 fold change; the y-axis shows −log10 P value. Significantly increased metabolites (FC > 1, P < 0.05) are in red; significantly decreased (FC < 1, P < 0.05) in blue; non-significant in gray. Note: In the PLS-DA plot, each point represents a subject sample (different colors/shapes correspond to different groups). In the correlation heatmap, red indicates a positive correlation and blue indicates a negative correlation.
